# Non-Linear Dynamical Classification of Short Time Series of the Rössler System in High Noise Regimes

**DOI:** 10.3389/fneur.2013.00182

**Published:** 2013-11-12

**Authors:** Claudia Lainscsek, Jonathan Weyhenmeyer, Manuel E. Hernandez, Howard Poizner, Terrence J. Sejnowski

**Affiliations:** ^1^Institute for Neural Computation, University of California San Diego, La Jolla, CA, USA; ^2^Howard Hughes Medical Institute, Computational Neurobiology Laboratory, The Salk Institute for Biological Studies, La Jolla, CA, USA; ^3^Indiana University School of Medicine, Indianapolis, IN, USA; ^4^Graduate Program in Neurosciences, University of California San Diego, La Jolla, CA, USA

**Keywords:** classification, Rössler attractor, non-linear dynamics, delay differential equations, electroencephalography

## Abstract

Time series analysis with delay differential equations (DDEs) reveals non-linear properties of the underlying dynamical system and can serve as a non-linear time-domain classification tool. Here global DDE models were used to analyze short segments of simulated time series from a known dynamical system, the Rössler system, in high noise regimes. In a companion paper, we apply the DDE model developed here to classify short segments of encephalographic (EEG) data recorded from patients with Parkinson’s disease and healthy subjects. Nine simulated subjects in each of two distinct classes were generated by varying the bifurcation parameter *b* and keeping the other two parameters (*a* and *c*) of the Rössler system fixed. All choices of *b* were in the chaotic parameter range. We diluted the simulated data using white noise ranging from 10 to −30 dB signal-to-noise ratios (SNR). Structure selection was supervised by selecting the number of terms, delays, and order of non-linearity of the model DDE model that best linearly separated the two classes of data. The distances *d* from the linear dividing hyperplane was then used to assess the classification performance by computing the area *A*′ under the ROC curve. The selected model was tested on untrained data using repeated random sub-sampling validation. DDEs were able to accurately distinguish the two dynamical conditions, and moreover, to quantify the changes in the dynamics. There was a significant correlation between the dynamical bifurcation parameter *b* of the simulated data and the classification parameter *d* from our analysis. This correlation still held for new simulated subjects with new dynamical parameters selected from each of the two dynamical regimes. Furthermore, the correlation was robust to added noise, being significant even when the noise was greater than the signal. We conclude that DDE models may be used as a generalizable and reliable classification tool for even small segments of noisy data.

## 1 Introduction

Electroencephalography (EEG) is a well studied and highly utilized tool for analyzing the brain activity of subjects in passive and active states. It is considered ideal for many studies because it is non-invasive and has the temporal resolution necessary to monitor cortical state changes. Due to the brain’s inherent non-linearity at cellular and mesoscopic scales (1), much emphasis has been placed on describing macroscopic scalp EEG waveforms as non-linear signals (2, 3). The identification of non-linear structure in human EEG has opened up a wide field of research for the application of non-linear dynamics to neurological waveforms, see (4–6). Subsequently, many studies have attempted to use non-linear techniques to analyze chronic neurological disease states including Alzheimer’s (7), epilepsy (8–12), Creutzfeld–Jacob (13), and Parkinson’s disease (14, 15). These studies have focused on quantifying the amount of non-linearity or complexity present in the EEG waveform using non-linear measures. Explicitly, given that the healthy EEG waveform is sparsely and sporadically non-linear (2), pathological states can be characterized by either increasing or decreasing non-linearity as measured by the correlation dimension, lyapunov exponent, or signal entropy. Unfortunately, these methods have difficulty when applied to non-stationary, quickly changing signals (7, 12). In this paper we present a new method for classifying non-linear, chaotic time series that have been constructed from similar dynamical systems with selected parametric differences. While the newly introduced method is meant to be applied to EEG time series, the present analysis was carried out on a simple dynamical system in order to provide better understanding of the method’s response to small, easily controlled changes in the underlying dynamical system. We hypothesized that this method would be able to differentiate time series that differ by a single underlying dynamical parameter based on dynamical properties observed in the signal itself. The method is well suited for real data as it does not suffer from the limitations of assumed stationarity and/or need for large data segments; issues that are commonly seen with traditional non-linear analysis techniques. Finally, our companion paper (Lainscsek et al., submitted) provides an in-depth, dynamical analysis of real Parkinsonian (PD), and healthy control (CO) EEG signals in order to identify and subsequently quantify differences in order to classify the EEG signals.

The Rössler system (16), composed of dynamic non-linear ordinary differential equations, was used to simulate time series for the present analysis. The system was chosen because it is low-dimensional and well studied in the field of non-linear analysis. Analysis of the generated Rössler data is presented in a proof-of-concept format whereby the underlying dynamics of a noisy, non-linear, chaotic system were used to differentiate between subjects whose defining feature was a single input parameter, *b*. In order to extend our analysis to real Parkinsonian and healthy control EEGs, we hypothesized that PD and CO subject EEGs would have different non-linear dynamical properties due to the underlying pathology of Parkinson Disease, e.g., dopamine depletion. Implicit in our hypothesis is the assumption that a chaotic, non-linear system has an underlying dynamical structure that can be quantified with non-linear analysis even though the structure may not be discernible by visual inspection or pattern recognition. The Rössler system was used to generate data series to gain a better understanding of DDE analysis on real data. We did not make any attempt to compare the simulated data with actual Parkinson or non-pathological EEG data, but rather assume that certain complex properties, the relevant features of the underlying dynamical structure, are present in both simulated and real data. Based on these conjectures, we designed an algorithm that is able to classify simulated Rössler waveforms in a noisy, chaotic system. Furthermore, the algorithm is able to correlate the output (classification) with the bifurcation parameter used to construct the waveform.

The paper is organized as follows. Section 2 introduces the simulated data from the Rössler system. Section 3 explains the DDE classification method and Section 4 shows the structure selection of good DDE models. Analysis and results can be found in Section 5. Section 6 is the discussion.

## 2 Rössler Data

**Figure 1:**
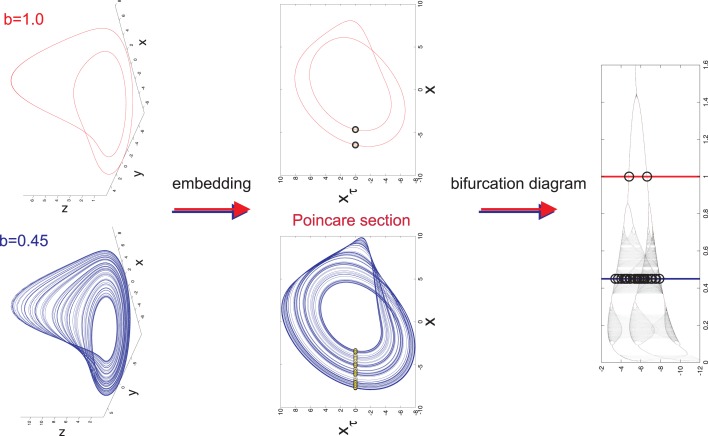
**Long term behavior of chaotic (*b* = 0.45) and simple (*b* = 1.0) attractors (top panel)**. Embeddings of *x*(*t*) in $xx_{\tau }$-plane that are used to generate Poincaré sections (middle panel). The intersections of the embedded time series with the line *x_τ_* = 0 are marked with yellow dots. The bifurcation diagram generated from the Poincaré section with *b* = 0.45 (blue line) and *b* = 1.0 (red line) marked to display the relationship between Poincaré sections and the bifurcation diagram (lower panel).

The Rössler system (16),

(1) $$\begin{array}{ccc} ẋ & =-y-z & \\ ẏ & =x+ay\\ ż & =b-cz+xz \end{array}$$

is a non-linear dynamical system that provides either complex or simple outputs depending on the parameters *a*, *b*, and *c* that are chosen. Integrating the dynamical equations with respect to time results in the formation of a 3-dimensional object known as an attractor. The attractor is a graphical representation of the longterm behavior of the dynamical system and is either chaotic or periodic. Periodic behavior is ascertained from the period-limit-cycle (Figure 1). The period-limit-cycle is the number of revolutions the attractor must make before converging on itself and repeating the cycle and is well defined for a simple system with true periodicity. A chaotic attractor does not have a well defined period-limit-cycle as the trajectories are chaotic and fail to converge upon previous loops.

In order to properly visualize the behavior of the attractor, a time series *x*(*t*) is extracted from each point (*x*(*t*), *y*(*t*), *z*(*t*)) on the attractor. *x*(*t*) is then embedded in the *xx_τ_*-plane such that *x*_τ_ = *x*(*t* − *τ*) is plotted against *x*(*t*). An embedding converts a single time series into a multidimensional object in an embedding space (17–20). The reconstructed attractor reveals basic properties (dimension, Lyapunov spectrum, entropy) of the true attractor of the system. It allows valuable information to be obtained about the dynamics of the system without having direct access to all the systems variables. There are two basic embeddings: delay and derivative embeddings. For a delay embedding the time series itself and its delayed versions are used to construct the embedding; for the derivative embedding the time series and its successive derivatives are used. Judd and Mees (21) introduced the idea of non-uniform embeddings for time series with components of multiple time-scales. From the embedding a Poincaré map is constructed in the *xx_τ_*-plane by plotting the value of *x*(*t*) every time it passes through a *specific line* in a *specific direction* (Figure 1). Here, the line is set to be at *x_τ_* = 0. The intersection points of this line with *x*(*t*) are the basis for the bifurcation diagram. The bifurcation diagram plots the Poincaré section generated for each bifurcation parameter *b* with *b* ranging from 0.01 to 1.6 (Figure 1). It is inferred from the bifurcation diagram that the behavior of the attractor is highly dependent on the value of the bifurcation parameter *b*. For example, *b* = 0.45 generates an attractor with chaotic behavior, however *b* = 1.0 generates an attractor with simple 2-period-limit-cycle behavior.

Data simulation is accomplished by allowing only the bifurcation parameter *b* to vary while setting *a* = 0.2 and *c* = 5.7. Random initial conditions are defined and the system is subsequently integrated with respect to time with an integration step size d*t* = 0.05. The data were then down sampled by using every fifth data point to have a similar number of pseudo periods as for the EEG data in the companion paper (Lainscsek et al., submitted). The system must be integrated for each value of *b* with each *b* producing its own attractor. For each attractor, a Poincaré section is generated and the values for *x*(*t*) are plotted on the bifurcation diagram for all *b*. Importantly, it does not matter whether *x*(*t*), *y*(*t*), or *z*(*t*) is chosen as the time series of interest because each individual time series contains all of the information needed to reconstruct the original dynamical system (19). Furthermore, the bifurcation diagrams constructed from *x*(*t*), *y*(*t*), *z*(*t*) and their embeddings *x_τ_*, *y_τ_*, *z_τ_* respectively, have the same dynamical properties. Thus, modeling of the data will be unaffected by the choice of time series or embedding.

**Figure 2:**
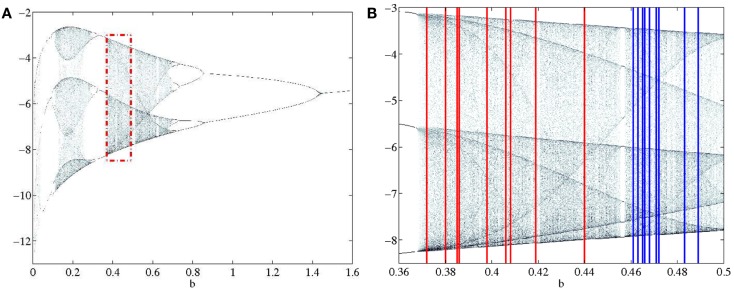
**(A)** Section of the bifurcation diagram from which the bifurcation parameters for all subjects were selected is outlined in red. **(B)** Enlarged view of bifurcation range with PD and CO ranges clearly separated. PD subjects range 0.37–0.44 and are shown in red. CO subjects range 0.46–0.49 and are shown in blue.

**Figure 3:**
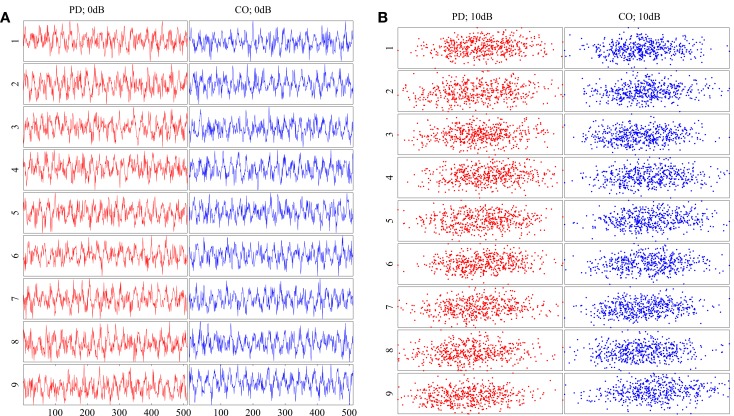
**(A)** Example Rössler time series for all subjects at SNR = 0 dB. **(B)** Embeddings of the time series in **(A)**. All time series and embeddings are very similar as there are no patterns that discriminate between classes. PD subjects are shown on the left column of each diagram in red. CO subjects are shown on the right column of each diagram in blue.

Two classes of data were generated, both in the chaotic range of the bifurcation diagram. Each class was given a non-overlapping range where the parameter *b* was the only variation between signals generated (Figure 2). Each class was composed of 9 subjects in order to emulate the data set presented in the accompanying paper (Lainscsek et al., submitted). Within the classes, each subject was given a unique *b* that fell within the range of the respective class. It was expected that Parkinson patients would have a wider range of variability than non-pathological subjects. In keeping with this idea, the simulated PD subjects were given a wider range for parameter *b*. This assumption was validated in our companion paper (Lainscsek et al., submitted) where real PD EEGs showed increased variability when compared with control EEGs. The Rössler signal produced was further constrained by the number of cycles per 1 s of data, forcing the Rössler to have similar frequency ranges to that of EEG. The sampling rate was set at 512 Hz so that each 1 s segment of data consisted of 512 points. For each subject, the time series *x*(*t*) was used to create 50 randomly selected data segments of 1 s duration by randomly selecting the data segments from an elongated signal. Prior to selecting the 50 data segments, the first 90,000 data points were discarded in order to remove the effects of initial conditions and isolate *b* as the major difference between subjects. Noise was added to the simulated signals to generate a signal-to-noise ratio (SNR) ranging between 10 and −30 dB, further imitating real EEG conditions while also providing additional information as to classification performance in noisy systems. Examples of the generated data and the respective embeddings are shown in Figure 3. Both the time series and the embeddings look very similar within and across classes.

## 3 Delay Differential Equations

Here we used delay differential equations (DDEs) in an attempt to classify simulated Rössler data from two separate distinct dynamical ranges based on the underlying dynamics of their respective signals. Since we use this data set as simulation data to better understand real EEG data in our companion paper (Lainscsek et al., submitted) we will call the two dynamical ranges PD and CO. A DDE is a generic non-uniform embedding (22) of a signal *x*(*t*) that relates the derivative of the signal ẋ(*t*) to the signal non-uniformly shifted in time *x_τ_* such that

(2) $$\begin{array}{ccc} ẋ & =f\left(x,x_{\tau_{1}},x_{\tau_{2}},\ldots x_{\tau_{n}}\right) & \\ & =a_{1}x_{\tau_{1}}+a_{2}x_{\tau_{2}}+a_{3}x_{\tau_{3}}+\ldots +a_{i-1}x_{\tau_{n}}\\ & \;+a_{i}x_{\tau_{1}}^{2}+a_{i+1}x_{\tau_{1}}x_{\tau_{2}}+a_{i+2}x_{\tau_{1}}x_{\tau_{3}}+\ldots +a_{j-1}x_{\tau_{n}}^{2}\\ & \;+a_{j}x_{\tau_{1}}^{3}+a_{j+1}x_{\tau_{1}}2x_{\tau_{2}}+\ldots +a_{l}x_{\tau_{n}}^{m} \end{array}$$

where *x* = *x*(t) and $x_{\tau_{n}}=x\left(t-\tau_{n}\right)$. The right side of Equation (2) can be expanded out to include many terms and non-linearities. Setting a limit on the number of terms and/or order of non-linearities allowed in the right-sided polynomial produces a low-dimensional DDE that is capable of capturing distinguishing dynamical features of the data. Since we are interested in classification, a low-dimensional DDE’s inability to entirely describe the original signal *x*(*t*) is not an issue. Our analysis was limited to two delays and monomials up to cubic non-linearities,

(3) $$\begin{array}{llll} ẋ & =f\left(x_{\tau_{1}},x_{\tau_{2}}\right)=a_{1}x_{\tau_{1}}+a_{2}x_{\tau_{2}}+a_{3}x_{\tau_{1}}^{2}+a_{4}x_{\tau_{1}}x_{\tau_{2}}+a_{5}x_{\tau_{2}}^{2}\; & & \;\\ & \;+a_{6}x_{\tau_{1}}^{3}+a_{7}x_{\tau_{1}}^{2}x_{\tau_{2}}+a_{8}x_{\tau_{1}}x_{\tau_{2}}^{2}+a_{9}x_{\tau_{2}}^{3}. \end{array}$$

The time delay τ_n_ in each term of the DDE ranged from 1 to 50 time-steps δ*t*, further increasing the number of model-delay combinations and signal estimating capacity. The derivative was computed numerically using a center derivative algorithm (23). The coefficients *a*_i_ were estimated numerically by a singular value decomposition (SVD) algorithm (24). The deviation of the model output $f\left(x_{\tau_{1}},x_{\tau_{2}}\right)$ from the signal derivative ẋ is henceforth called the error ρ of the model and is calculated with mean-squared error estimation $\rho =\sqrt{\sum {\left(ẋ-f\left(x_{\tau_{1}},x_{\tau_{2}}\right)\right)}^{2}}$. ρ can be minimized by optimizing the structure of the DDE according to the dynamics and the delays according to the time-scales in the data. Time-shift scaling adds frequency information to the DDE model such that linear DDE terms are related to the linear frequency content of the EEG signal and non-linear DDE terms are related to frequency coupling (22). Only models of 2 or 3 terms were considered and all *a*_i_ not included in Equation (3) were set to zero. The error-coefficient space describes a particular DDE’s ability to model a specific signal. For our purposes we did not attempt to exactly model or recreate Rössler signals from the DDEs. The DDE models were used for classification (22, 25, 26), giving an output that corresponded to either a PD or CO input class. The primary objective of the structure selection was to obtain DDE models that maximally separate CO and PD subjects. Ideally the outputs are completely separated such that there is no overlap of outputs between the two groups. In order to obtain models that best separated the groups, a repeated random sub-sampling validation scheme (27) was implemented.

## 4 Supervised Structure Selection

### 4.1 Training

Repeated random sub-sampling validation (27) is a method of training and testing on a single dataset that was employed to prevent over-fitting of a model to the dataset, thereby increasing the generalizability of the experimental findings. First each class was partitioned into subgroups containing six subjects and three subjects. The group of six subjects from the PD class and the six subjects from the CO class were chosen to act as the training data. This left three subjects from each group on which to test the trained models. The grouping was repeated 84 times so that all possible combinations were used for training and testing. DDE selection was performed on training subjects for each of their respective 50 1 s segments of data. The window length on which the DDE outputs were computed was set to 1 s, 1 window per data segment. 600 data windows (2 classes × 6 subjects × 50 windows) were computed for each structure selection performed. The first 300 windows, *i* = 1:300 computed outputs for the six control subjects, and *i* = 301:600 computed outputs from the six subjects from the PD group. In DDE selection, a model-delay pair was fit to a signal of interest using an SVD algorithm that numerically computed the coefficients using a least-square-error estimation. The calculated coefficients and error are placed into the matrix **A***_i,j_*,

(4) $$A_{i,j}={\left(a,\rho \right)}_{i,j}=\left(\begin{array}{cccc} a_{1,1} & a_{1,2} & a_{1,3} & \rho_{1}\\ \vdots & \vdots & \vdots & \vdots \\ a_{300,1} & a_{300,2} & a_{300,3} & \rho_{300}\\ a_{301,1} & a_{301,2} & a_{301,3} & \rho_{301}\\ \vdots & \vdots & \vdots & \vdots \\ a_{600,1} & a_{600,2} & a_{600,3} & \rho_{600} \end{array}\right)$$

Given that we have 50 DDE models restricted to 2 or 3 terms and time constants τ_1,2_ that range from 1 to 50, there are a total of 103100 model-delay pairs. Table 1 lists all the DDE models. Note that, e.g. the DDE models $ẋ=a_{1}\;x_{\tau_{1}}+a_{2}\;x_{\tau_{1}}\;x_{\tau_{2}}$ and $ẋ=a_{1}\;x_{\tau_{2}}+a_{2}\;x_{\tau_{1}}\;x_{\tau_{2}}$ are the same with exchanged delays τ_1_ and τ_2_. Therefore only the first of these two models was used. All such redundant DDE models were omitted. Explicitly, 103100 **A***_i,j_* matrices were generated where **A***_i,j_* contained *a*_1_, *a*_2_, *a*_3_, and ρ computed for each of the 600 windows. Provided the coefficient-error matrix had definite separation between the two classes in the coefficient-error space, it was possible to estimate a weight matrix **W***_j_* using SVD such that **A***_i,j_* ⋅ **W***_j_* = **S***_i_* where **S***_i_* maps the outputs for CO and PD subjects to opposite sides of a predefined hyperplane,

(5) $${\text{A}}_{i}\;\cdot \;{\text{W}}_{i}={\text{S}}_{i}=\left\{\begin{array}{cc} 1\; & i\in CO\left(i\leq 300\right)\\ -1\; & i\in PD\left(i>300\right)\\ \; \end{array}\right.$$

**Table 1:** **Two- and three-term models**.

Model no.	$a_{1}x_{1}$	$a_{2}x_{2}$	$a_{3}x_{1}^{2}$	$a_{4}x_{1}x_{2}$	$a_{5}x_{2}^{2}$	$a_{6}x_{1}^{3}$	$a_{7}x_{1}^{2}x_{2}$	$a_{8}x_{1}x_{2}^{2}$	$a_{9}x_{2}^{3}$	Model type
1	*x*	*x*								S, L
2	*x*		*x*							1
3	*x*			*x*						
4	*x*					*x*				1
5	*x*						*x*			
6		*x*	*x*							
7		*x*		*x*						
8		*x*				*x*				
9		*x*					*x*			
10			*x*	*x*						
11			*x*		*x*					S
12			*x*			*x*				1
13			*x*				*x*			
14				*x*		*x*				
15				*x*			*x*			
16						*x*	*x*			
17						*x*			*x*	S
18							*x*	*x*		S
19	*x*	*x*	*x*							
20	*x*	*x*		*x*						S
21	*x*	*x*				*x*				
22	*x*	*x*					*x*			
23	*x*		*x*	*x*						
24	*x*		*x*		*x*					
25	*x*		*x*			*x*				1
26	*x*		*x*				*x*			
27	*x*			*x*		*x*				
28	*x*			*x*			*x*			
29	*x*					*x*	*x*			
30	*x*					*x*			*x*	
31	*x*						*x*	*x*		
32		*x*	*x*	*x*						
33		*x*	*x*			*x*				
34		*x*	*x*				*x*			
35		*x*		*x*		*x*				
36		*x*		*x*			*x*			
37		*x*				*x*	*x*			
38			*x*	*x*	*x*					S
39			*x*	*x*		*x*				
40			*x*	*x*			*x*			
41			*x*		*x*	*x*				
42			*x*		*x*		*x*			
43			*x*			*x*	*x*			
44			*x*			*x*			*x*	
45			*x*				*x*	*x*		
46				*x*		*x*	*x*			
47				*x*		*x*			*x*	S
48				*x*			*x*	*x*		S
49						*x*	*x*	*x*		
50						*x*	*x*		*x*	

An “*x*” denotes that the term a* is non-zero. The different types of models are: “L,” linear; “S,” symmetric; “1,” single delay DDE. All other DDEs are non-linear and have two non-interchangeable delays.

The weight matrix forces the CO class to the positive side of the hyperplane and the PD class to the negative side of the hyperplane providing a 2-dimensional mapping of the separation. Upon completion of training, 103100 **W***_j_* were computed.

### 4.2 Testing

The DDE outputs for the six testing subjects were computed and put into a matrix **T***_k,j_* using the SVD and least-square-error scheme previously defined. A coefficient-error matrix **T***_k,j_* was computed for each of the 103100 model-delay pairs with each matrix containing the information for all 300 windows (2 classes × 3 subjects × 50 windows). The outputs for the CO class were placed into *k* = 1:150 and the outputs for the PD class were placed in *k* = 151:300. The previously computed weight matrices **W***_j_* were tested against **T***_k,j_*. Thus,

(6) $${\text{T}}_{k,j}\cdot {\text{W}}_{j}=d_{k},$$

where *d_k_* is the positive or negative distance from a predefined hyperplane for each window. *d_k_* provided the information necessary to generate a receiver operating characteristic (ROC). The model-delay pair classification capability was assessed by computing the area under the ROC curves, *A*′ (28). A ROC curve is a plot of the cumulative distribution function of the CO class against the cumulative distribution function of the PD class [see (28), p. 173 for exact definitions]. To compute the area *A*′ under the ROC curve [following the approach introduced in Ref. (28)], we ranked the distances from the hyperplane *d* from the largest positive value to the most negative value. Let *r_i_* be the rank of the *i*th control subject point. The area under the ROC curve is approximated by

(7) $$A\prime =\frac{S_{0}-n_{0}\left(n_{0}+1\right)∕2}{n_{0}n_{1}}$$

where *S*_0_ is the sum of the ranks of the control subject points, *n*_0_ the number of CO subjects, and *n*_1_ the number of PD subjects. For each set of training and testing, a total of 103100 *A*′s were generated. As previously mentioned, the grouping process was repeated 84 times and the 84 *A*′s found for each model-delay pair were averaged in order to find the best performing model-delay pairs. Importantly, 84 **W***_j_* were also generated for each model-delay pair and the W*_j_* corresponding to the best performing model-delay pair were averaged. The averaged-best-performing model-delay pair and weight vector were then tested on the data set.

## 5 Numerical Experimentation and Results

### 5.1 Additional data and new subjects

**Figure 4:**
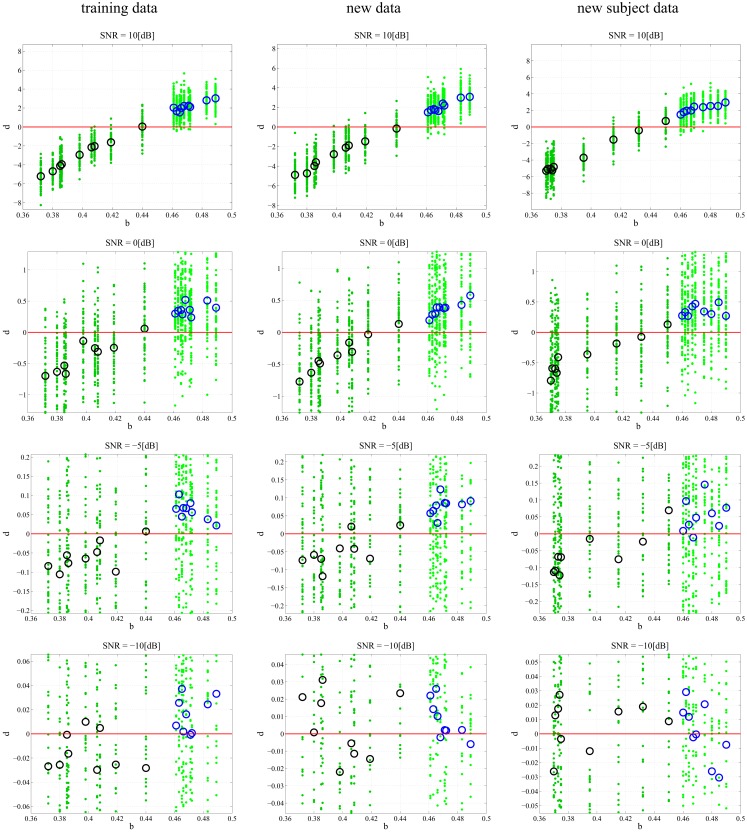
**Classification of different data sets at different SNRs**. Each subplot maps the output *d* value against the input *b*. Small dots signify the classification of each, individual trial of the 50 trial set. The PD subjects (dark green dots) have more negative *d* values and the CO subjects (light green dots) have more positive *d* values with zero selected as the hyperplane and plotted as a continuous red line. The larger circles represent the mean *d* for over all 50 trials for each subject; black circles correspond to PD and blue circles correspond to CO groups. The performance of the DDE classification algorithm on the training data, new data, and new subject data is plotted from left to right respectively. SNR ranges 10:−10 dB and decreases from top to bottom. The system shows noise invariance up to −5 dB after which the ability to discriminate between the two classes decreases dramatically. At high SNR the bifurcation parameter *b* is linearly correlated to the output *d*.

In any data derived classification technique, there is always a risk of over-fitting (29) to a training data set such that there is exceptional performance on the data set in question but poor generalization when additional data sets are tested. We chose to employ repeated random sub-sampling validation (30) to ensure that our classification did not over fit the data. We validated this assumption through two separate experiments that were meant to either prove or disprove an over-dependence on the training data. First, every subject had an additional 50 random 1 s data segments taken from its attractor: additional data. The previously found best model-delay pair and weight matrix were tested against coefficient-error matrices found via SVD for the new data sets (Section 4). The resulting *d* is shown graphically (Figure 4) with varying degrees of noise. Second, we generated an entirely new subject pool that was held to the same constraints as the original Rössler data with each class, PD and CO, given nine additional subjects. The data sets were based on the bifurcation parameter *b* where subjects of the PD and CO classes fell within the previously defined ranges: new subject data. Again, the top performing model-delay pair and weight matrix (Section 4) were used to classify the newly created subjects (Figure 4). The newly generated subject outputs show a clear relationship between the bifurcation parameter *b* and the distance *d* from the hyperplane. The addition of new subjects to each class and the continued high performance of the classification scheme indicates that provided the underlying dynamics of two classes of subjects are different, it is possible to differentiate between the two classes. Moreover, a subject’s distance from the hyperplane provides a meaningful measure of how different its underlying dynamics are from other subjects both within and outside of its own class.

### 5.2 Noise

In order to understand how the repeated random-sub-sampling DDE selection algorithm would perform against real EEG data it was employed in regimes with varying levels of noise. Specifically, white noise was added to the Rössler data until signal to noise ratios (SNR) of 10, 5, 0, −5, −30 dB were attained. Biasing of the classification scheme for increased performance was avoided by retraining and retesting on the newly created noisy data affording a measure of noise invariance. Each SNR implementation went through training and testing in order to generate a different average-best-model-delay pair and weight matrix. This means we re-trained and re-tested at each level of SNR. The distance *d* from the predefined hyperplane was calculated for each trial of each subject along with the mean over all 50 trials. The resulting 50 *d*’s and mean *d* were plotted with respect to the bifurcation parameter given to each subject at the various SNRs (Figure 4). Noise was applied to the initial training data set, new data, and to the new subject data. While the inclusion of extreme amounts of noise does appear to make the classification task more difficult, especially for outlier bifurcation parameters that are situated near the range of the other class, it is still possible for the classification scheme to perform at a high level. Importantly, the bifurcation parameter *b* is correlated to the distance *d* from the hyperplane (Table 2). The linear correlation indicates that the classification scheme is identifying predominant underlying dynamical differences of the system and is able to quantify these differences in a meaningful way. Furthermore, the ability to classify signals at an SNR of −5 dB, a situation where there is more noise than signal, is an indication of noise invariance within the classification scheme.

**Table 2:** **Correlation coefficients between the dynamical parameter *b* and the distance *d* from the hyperplane for different levels of noise**.

SNR	Training data	New data	New subjects	SNR	Training data	New data	New subjects
10	1.00	1.00	1.00	−15	0.61	0.10	0.04
5	1.00	0.99	0.99	−20	0.61	−0.09	−0.08
0	0.97	0.99	0.97	−25	0.61	0.21	−0.28
−5	0.88	0.92	0.86	−30	0.66	0.25	−0.00
−10	0.68	−0.02	−0.15	

### 5.3 Data shuffling

**Figure 5:**
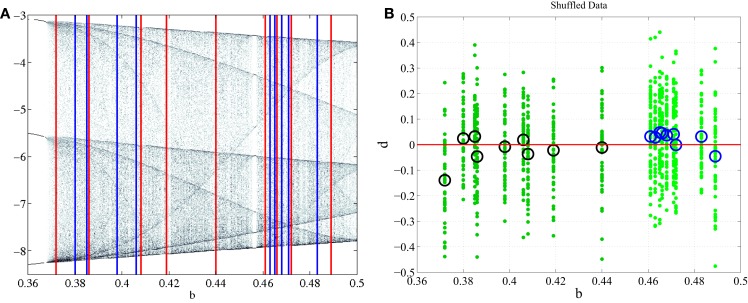
**Subject shuffling**. **(A)** PD subjects, red, and CO subjects, blue, are randomly placed in the bifurcation range without any separation between their ranges. **(B)** After retraining and retesting, *b* is plotted against *d*. There is no longer a correlation between input *b* and output *d* of the classification system.

Non-linear classification of pathological and healthy states assumes that there is a quantifiable dynamical difference between the two states. When comparing PD and CO classes, it is expected that the dynamical features isolated by DDE analysis have meaning such that all PD subjects fall into a specific feature set and all CO subjects fall into a different feature set. The Rössler system simplifies the identification of the feature set by making the long term behavior of the system dependent on a single feature, the bifurcation parameter *b*. Grouping bifurcation parameters into classes that are made up of specific ranges of *b* is valid only if all *b* values in a given range correspond to the particular class at the output of the DDE system. Shuffling the data, whereby the ranges of PD and CO subjects are no longer separable (Figure 5A), provides evidence as to how our classification technique will perform if there are no dynamical differences between groups being compared. After shuffling the data, model-delay pair selection and testing was performed (Section 4). It can be seen from the graphical results of the best performing model-delay pair (Figure 5B) that classes with no dynamical differences will not be separable by a hyperplane, regardless of weight matrix or model-delay pair selected. Thus, if classes of EEG waveforms have highly overlapping feature sets, such that they are inseparable in dynamical feature space, it is expected that dynamical analysis will be unable to classify the waveforms into separate groups.

**Figure 6:**
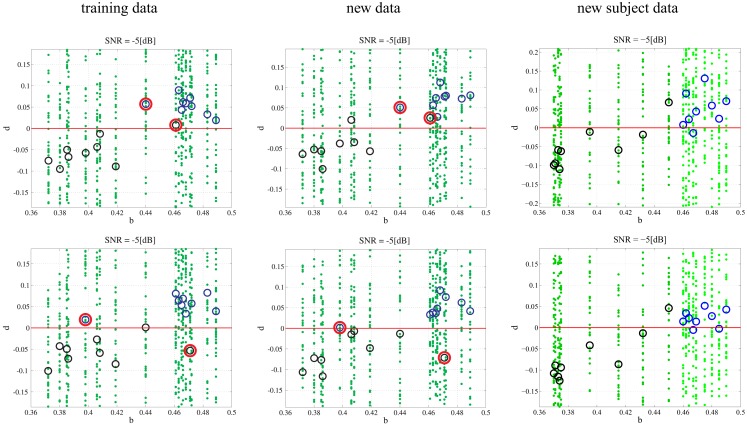
**Single outlier in each class: in the upper row the subject with the largest bifurcation parameter in the PD class and the subject with smallest bifurcation parameter in the CO class were switched (outlined in red)**. In the lower row the subject with a bifurcation parameter closest to the mean of the bifurcation range for each class was switched (outlined in red). The green dots refer to the 50 single trials in each subject while the circles denote the mean values (black for PD and blue for CO).

Additionally, it is important to investigate the consequences of subjects with outlier bifurcation parameters and their effect on training and testing. In order to observe the effects of large outliers, a single subject from each class was switched into the opposite class and training and testing was performed on the training data, additional data, and new subject data. The switching of subjects was performed in two ways. First, the subject with the largest bifurcation parameter in the PD class and the subject with smallest bifurcation parameter in the CO class were switched. This has the effect of lengthening the parameter range for each class such that the ranges now overlap. Second, an outlier was created for each class by selecting and switching the subject with a bifurcation parameter closest to the mean of the bifurcation range for each class. The newly created subjects were then labeled to the opposite class. Both experiments were performed at −5 dB. Overlapping the classes (Figure 6) by switching the largest parameter in the PD class and the smallest parameter in the CO class does not appear to have a significant affect on classification unless the bifurcation parameters fall within the crossover range. Indeed, a lack of true separation between bifurcation parameter ranges would be expected to make classification within the crossover range exceedingly difficult. Extrapolation to new subjects provides a similar result where the subjects falling into the crossover range are difficult to classify. In Figure 6 the classification algorithm is shown to be robust to a single gross outlier. The classification scheme is still able to classify the subject properly within the training data by calculating the PD subject to be a negative distance from the hyperplane and the CO subject to be a positive distance from the hyperplane. Thus, even with wrong labels, the model is able to find the right dynamics and sparse outliers do not appear to have a significant effect. Testing on the additional data shows similar results (Figure 6). Finally, testing on the new subjects that retained the initial bifurcation ranges (Section 2) shows great classification. Again, the correct classification of additional subjects that fall within the previously defined separable parameter ranges provides strong evidence that the algorithm is robust to single gross outliers.

### 5.4 Combining models

Up to this point, classification has been performed with a single model-delay pair and a single weight matrix and has yielded good performance. As is the case with other analytical techniques, it should be possible to increase performance by combining well performing model-delay pairs. Horizontally concatenating previously calculated **A***_i,j_* for well performing model-delay pairs,

(8) $${\text{C}}_{i,N}=\left({\text{A}}_{{i,j}_{1}},{\text{A}}_{{i,j}_{2}},\ldots ,{\text{A}}_{{i,j}_{n}}\right)$$

leads to a model-delay pair combination of carefully selected terms. *N* is defined as the number of non-zero terms in a single row of the horizontally concatenated matrix. The number and type of model-delay pairs chosen was limited in order to elicit the best performance without over-fitting the training data set. Initial training and testing calculated the top performing model-delay pairs based on averaged *A*′s (Section 4). The top performing pairs were combined such that if the model combination was limited to five model-delay pairs then only the top five performing model-delay pairs were used for the combination classification. Combinations of up to 30 model-delay pairs were implemented in order to search for increased performance in high noise regimes. A new weight matrix **W***_N_* is generated by constraining,

(9) $${\text{C}}_{i,N}\cdot {\text{W}}_{N}={\text{S}}_{i}=\left\{\begin{array}{cc} 1\; & i\in CO\left(i\leq 300\right)\\ -1\; & i\in PD\left(i>300\right)\\ \; \end{array}\right.$$

**Figure 7:**
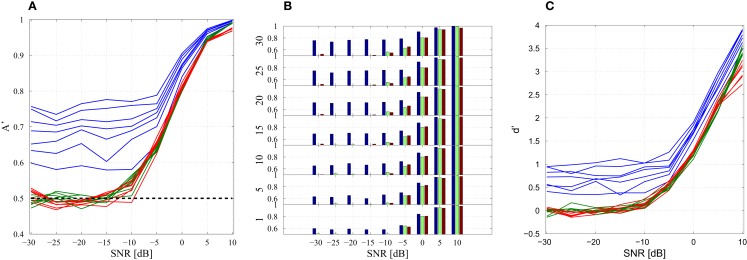
**Combination of models**. **(A)** Line of plot of *A*′ with respect to SNR. Training data set performance is in blue, new data is in green, and new subject data is in red. Each model-delay pair number: 1, 5, 10, 15, 20, 25, 30 is plotted with a separate line with each data set having seven lines. The black line *A*′ = 0.5 indicates 50% probability of correct classification. **(B)** Bar graph of classification performance on each data set. Each model-delay pair combination is plotted separately. **(C)** Sensitivity index *d*′ at various levels of SNR for model-delay combinations of length 1, 5, 10, 15, 20, 25, and 30.

Importantly, there are still 600 data windows in the concatenated matrix and the training of the weight matrix **W***_N_* is identical with the previous technique (Section 4). The performance of different model combinations at varying SNRs are shown in Figures 7A,B with both plots displaying the same information in different formats. At high SNRs, a single model-delay pair already provides close to maximum performance (*A*′ = 1.0) for all data cases and thus increasing model number is not beneficial. As expected, the classification performance decreases when introducing additional data and new subjects, however, the performance remains well above chance (*A*′ = 0.5) for all data sets down to and including −5 dB. Classification capabilities decrease significantly after −5 dB regardless of model combination. Increasing model number appeared to only increase the classification of the training data set with negligible effects, good or bad, on the additional data and new subjects. Further displaying the discrimination capabilities of the present algorithm, the sensitivity index *d*′ (31) was calculated (Figure 7C). The sensitivity index *d*′ (not to be confused with the distance from the hyperplane *d*) shows similar behavior to the traditional ROC in that the signal is easily discerned at high SNR but SNRs lower than −5 dB make signal discrimination much more difficult.

## 6 Discussion

Analyzing the Rössler system while only varying the bifurcation parameter allowed for a simplified proof of the capabilities of the DDE classification scheme to identify underlying dynamical differences between waveforms with many similarities in the time and spectral domain. The continued classification of dynamical systems in high noise regimes provides further support to the argument that the method presented here is applicable to the classification of measurements taken from high noise systems, e.g., EEG. The Rössler system is one of many possible oscillators (e.g., the Lorenz system, Colpitts oscillator, or neural mass models) that may be used for this analysis. However, the primary reason for using the Rössler system was the ease of varying specific dynamical parameters without greatly changing the observable time series. As such, the results presented in this paper are meant as a proof-of-concept of the DDEs ability to classify a system based on its underlying dynamical parameters using only the information obtained from an observable time series. Additionally, this experiment made no attempt to analyze or classify coupled or synchronized systems. The complete understanding of the underlying dynamics of a neurological system and its pathologies will require additional analysis of large scale coupling and is considered a future direction of this project. However, it should be emphasized that the classification technique presented here has been shown to be highly correlated to Parkinson’s disease pathology and furthermore to the gradation of pathological severity in our companion paper (Lainscsek et al., submitted).

Perhaps the most profound finding of this experiment is not the ability to classify Rössler signals into their respective classes, but rather the ability to linearly correlate the bifurcation parameter *b* to the output *d* (Figure 7 and Table 2). Indeed, the output *d* appears to grade the input parameter *b* of the dynamical system. Grading is step beyond binary classification, providing a means to differentiate between subjects within a class and objectively rate the degree of difference. Additionally, as the bifurcation parameter of a specific class takes on values close to the range of the other class, it becomes increasingly difficult for the classification scheme to correctly classify which class the waveform originated from. This is quite similar to the onset of chronic pathological states that are indolent at the beginning and tend to get progressively worse with time. Thus, distance from the hyperplane may be correlated with disease level when applying the classification technique to pathological EEG. Extrapolating the grading system for use on chronic disease states requires that the distance *d* be mapped against a known measure, e.g., UPDRS scores. The correlation between the DDE outputs of Parkinson finger-tapping movement data and UPDRS scores was shown previously (26). Whether or not *d* will continue to correlate with known units of measure for pathological states is an open question with many profound implications.

It is interesting that at very low SNRs, increasing the model number serves only to increase the classification performance of the training data set (Figures 7A,B and Table 2). While similar to what would be expected in over-fitting, the overall trend is different. With decreasing SNR there is an expected monotone decrease in classification performance. However, the classification performance on the new data and new subjects does not appear to be related to the number of model-delay pair combinations. If this was simply a case of over-fitting, we would expect decreased classification performance when increasing the number of model-delay pairs. Furthermore, the performance should be worse at each increment of total model-delay pair number when classifying new subjects, but this is not the case (Figures 7A,B). Increasing the number of model-delay pair combinations appears to only improve classification of the initial training data in high noise regimes, with the increased number of model-delay pairs allowing the classification scheme to “lock-in” on the data on which it was trained without affecting performance on new data. As previously stated, analyzing only nine subjects for each class results in an incomplete dynamical description of the unknown underlying dynamical system. It is conceivable that increasing the amount of training subjects and data would provide enough information such that increasing model number would be useful when attempting to classify new data. It is important to be mindful that training and testing of this data driven technique was only performed on 18 subjects taken from two distinct 9 subject classes. Training on such a limited data set is unlikely to permit complete extrapolation of the underlying dynamics of a given class of data. Yet, when taking additional data from the training subjects and even with the inclusion of entirely new subjects restricted to the defined classes the classification technique was still able to perform well above chance at SNRs as low as −5 dB. The inclusion of more subjects would be very computationally intense and is beyond the scope of paper, but it is likely the more robust the training, the greater the performance, and generalizability of the classification system.

Applying the DDE classification method toward the differentiation of human brain states will require that it is capable of recognizing signals that vary in many different dynamical features rather than a single parameter. While there may be many dynamical differences between pathological and healthy control states, these differences may be small, making the resolution of the classifying scheme very important for correct classification. Ideally, variance in an increased number of dynamical features will offset the small differences between features within the brain states and permit correct classification. Additionally, some feature differences may simply arise due to normal variation between healthy individuals. Any classification scheme meant to differentiate pathology from healthy states will need to perform a selective search for relevant dynamical features. Thus, for the DDE to correctly classify pathological and normal EEG states, it will be necessary for the selected model-delay pairs to isolate only those features that are relevant to a specific pathology. Such features are unknown at present and may prove difficult to elucidate. However, the implementation of the random sub-sampling validation on pathological and non-pathological EEG waveforms may allow for the identification of unknown dynamical differences without any direct knowledge of processes that generated the differences, providing a classification scheme that is able to both diagnose and grade pathology based on non-invasive measurements.

## 7 Conclusion

This manuscript outlines a new dynamical approach to time series classification. In order to describe the capabilities of delay differential equations to classify dynamical differences, two classes differing only in a single dynamical parameter were used for time series construction. The Rössler system was chosen for this analysis because of its ease of implementation and the ability to isolate and change a single dynamical parameter. Method testing and validation was performed using repeated random sub-sampling in order to find model-delay pairs and a weight matrix that behaved well across the entire data set, regardless of which subjects were used for training and testing. Additional data and additional subjects were created and used to test the previously computed best model-delay pair and weight matrix in order to see how generalizable the algorithm was to data generated from an identical non-linear dynamical system as well as data generated from a parameter that fell within one of the previously outlined classes. In order to further emulate realistic conditions, high levels of noise were added to the simulated data and the method was shown to be noise invariant up to −5 dB. Finally, data shuffling was implemented to show that the classes of data must be separated dynamically in order for any type of classification to occur with the DDE method. We conclude that DDEs are able to identify and classify small changes in underlying dynamical systems that are not immediately recognizable in the observable time series. Such methods may prove to be extremely useful in the classification of time series observed in real world situations.

## Conflict of Interest Statement

The authors declare that the research was conducted in the absence of any commercial or financial relationships that could be construed as a potential conflict of interest.
